# Becoming a Rapid Shooter in a Game Using Embodied Electrical Muscle Stimulation: Development and Usability Study

**DOI:** 10.2196/69330

**Published:** 2025-10-22

**Authors:** Jihwan Kim, Mingyu Kang, Jejoong Kim, Kwanguk (Kenny) Kim

**Affiliations:** 1 Department of Computer Science, Hanyang University, 222 Wangsimni-ro, Seongdong-gu, Seoul, 04763, Republic of Korea, 82 222202319; 2 Department of Psychology, Duksung Women's University, Seoul, Republic of Korea

**Keywords:** electrical muscle stimulation, embodied interaction, human augmentation, sense of agency, serious game

## Abstract

**Background:**

Electrical muscle stimulation (EMS) systems enhance human capabilities, such as reaction time, by inducing preemptive muscle contractions. One of the key challenges for EMS applications is preserving the user’s sense of agency, and it is defined as a subjective experience of initiating and controlling one’s actions. Prior research highlights the importance of the “sweet spot”—a balance between sense of agency and preemptive gain—for effective EMS use. However, most prior studies have focused on simplistic tasks, leaving a gap in understanding how the sweet spot functions in complex and ecological game scenarios. Moreover, the potential benefits of a personalized approach were not explored.

**Objective:**

This study examines the effects of embodied EMS on performance and sense of agency in a serious-games–based pistol-shooting scenario. Additionally, we investigated the effects of personalization in the same game scenario.

**Methods:**

Two studies were conducted. Study 1 identified the optimal EMS timing (“sweet spot”) to improve response time while preserving agency. A total of 13 participants completed a visual stimulus-response task after EMS calibration. Participants performed 150 right-button clicks on a target using an EMS-equipped mouse, with EMS timings ranging from −200 to +100 ms. An agency questionnaire followed each trial. Logistic regression was used to identify the sweet spot. Study 2 applied the findings of Study 1 to a pistol-shooting game. A total of 10 new participants were recruited. Before gameplay, individual sweet spots were measured for the individually embodied EMS condition. During the game, participants navigated to a target location, distinguished enemies from hostages, and shot enemies. After a practice session, participants completed the game under 4 counterbalanced conditions: averagely embodied EMS (used the average sweet spot value from Study 1), individually embodied EMS (used each participant’s own sweet spot value), immediate EMS (stimulation at a target onset timing), and no EMS.

**Results:**

Study 1 identified a sweet spot that significantly improved reaction time while preserving agency. Logistic analysis showed an average sweet spot of 46.8 ms and individual variability across participants. Study 2 confirmed these findings in a pistol-shooting game. Both averagely and individually embodied EMS significantly reduced reaction times compared to no EMS (*P*=.03 and *P*=.001, respectively), without compromising agency. Individually embodied EMS yielded additional benefits. In the slower than average group, the sense of agency in the individually embodied EMS condition showed an additional benefit that was not observed in the averagely embodied EMS condition (*P*=.003 and *P*=.095, respectively).

**Conclusions:**

The findings indicate that embodied EMS enhances performance in complex game scenarios while maintaining users’ sense of agency, with individualization yielding additional benefits. These results extend prior evidence to more complex game contexts and provide valuable implications for the personalized design of EMS systems in sports training, rehabilitation, and serious gaming.

## Introduction

### Background

Technologies for augmenting human capabilities have advanced significantly with the development of electrical muscle stimulation (EMS). EMS enhances motor performance and reaction speed by inducing muscle contractions through externally applied electrical impulses [1-5]. A key challenge in EMS application is preserving the user’s sense of agency—the subjective perception of control over one’s actions—while achieving performance gains [6,7]. Prior studies have shown that carefully timed EMS can improve reaction performance without compromising agency, identifying a precise stimulation window known as the “sweet spot” [5,6]. However, most existing studies have used simplistic motor tasks, limiting the understanding of EMS in more realistic contexts [8,9]. Furthermore, although embodied EMS can yield a sweet spot, its personalization has not been explored, despite agency being a subjective experience [10-13]. To address these gaps, we investigate the effects of embodied EMS in a pistol-shooting game requiring rapid decision-making and response execution. We also assess the benefits of individualized EMS configurations.

The remainder of this paper is organized as follows: The *Methods* section presents details on the 2 experiments conducted. The *Results* section presents the results of both experiments. The final section discusses the results and offers future research directions.

### Previous Studies on EMS Applications

EMS generates electrical impulses that induce muscle contractions [14], typically delivered through electrodes placed on the skin near target muscles [10,15]. Compared to motor-based devices, such as exoskeletons, EMS is significantly more wearable [2], making it suitable for force feedback in augmented and virtual reality applications [16]. When combined with haptic instruction systems requiring rapid and precise movements [3,15-18], EMS has proven effective.

EMS is generally used to enhance physical capabilities. For example, the “Wired Muscle” system preemptively stimulates muscles to improve reflexes in physical tasks [19], although “Stimulated Percussions” guides hand movements in rhythmic tasks, enhancing timing and coordination in musical performance [20]. EMS also improves speed and accuracy in physical actions. The electrical head actuation system enables EMS-based control of head orientation, extending applications beyond the limbs to support gaze-guided selection and immersive head movement [15]. “Affordance++” guides limb movements in tasks such as shaking a spray can, reducing effort and training requirements [21], whereas “Pose-IO” directs wrist movements for accurate gesture-based communication without visual feedback [22]. A back-of-hand actuation system improves finger dexterity by enabling refined, independent control of finger joints [3].

Some systems use EMS to enhance immersion. “Paired-EMS” stimulates antagonistic muscle pairs to provide realistic, stable force feedback in high-intensity VR scenarios, improving user comfort and immersion [17]. “BioSync” synchronizes EMS across users, allowing shared kinesthetic experiences in real time [23]. “ErgoPulse” uses real-time biomechanical simulations to estimate muscle forces and joint angles, guiding lower-limb stimulation during virtual interactions such as walking or jumping [24]. EMS for walls and heavy objects simulates resistance during interaction with massive virtual structures, increasing realism in virtual environments [16,25]. A key advantage of EMS is its ability to reduce reaction time, making it particularly effective for applications requiring rapid responses [1,25]. The details of previous studies are summarized in Table 1.

**Table 1.** Previous studies using electrical muscle stimulation (EMS).

Author; year	Purpose	Target	Participants	Performance metrics
Kruijff et al [25], 2006	To create pseudo-haptic feedback using EMS	Biceps or brachioradialis	7 (user evaluation)	Haptic realism, perceived resistance
Lopes et al [22], 2015b	To communicate object-specific gestures using EMS	Flexor digitorum, flexor carpi radialis, extensor digitorum, flexor digitorum profundus, biceps brachii	12 (user evaluation)	Understanding the meaning of induced motion, helpfulness, visual affordance
Lopes et al [21], 2015a	To improve limb position awareness through EMS-based proprioception	Extensor digitorum, flexor digitorum superficialis	10 (system validation), 12 (user evaluation)	Angular posture accuracy, subjective experiences
Ebisu et al [20], 2016	To guide rhythmic movement for music training using EMS	Extensor carpi radialis, gastrocnemius, brachioradial muscle	12 (user evaluation)	Sound timing, rhythm accuracy
Nishida et al [19], 2017	To enable faster kinesthetic synchronization	Extensor digitorum	N/A^a^	Reaction time, subjective reaction fluency
Nishida and Suzuki [23], 2017	To create shared motion experience between users via synchronized EMS	Extensor digitorum	5 (design validation), 5 (formative study 1), 6 (formative study 2), 1 (user experience)	Synchronization quality, subjective response
Lopes et al [16], 2017	To simulate physical resistance in virtual reality using EMS on arm/shoulder	Extensor digitorum, infraspinatus, extensor carpi ulnaris, teres major/minor	13 (design validation), 6 (user evaluation)	Preference, realism, consistency, impermeability
Kasahara et al [6], 2019	To accelerate reaction time using sense of agency preserving EMS	Flexor digitorum profundus	12 (formative study),12 (user evaluation)	Sense of agency, reaction time
Kasahara et al [5], 2021	To evaluate motor adaptation by EMS	Flexor digitorum profundus	18 (formative study),17 (user evaluation)	Sense of agency, reaction time
Takahashi et al [3], 2021	To improve finger dexterity using EMS on the back hand	Extensor muscles	9 (user evaluation)	Movement ratio between finger joints, calibration time
Tanaka et al [15], 2022	To control head orientation via EMS for hands-free virtual reality interaction	Splenius capitis, splenius cervicis, sternocleidomastoid	7 (system validation), 8 (user evaluation)	Head movement accuracy, contribution of head actuation, enjoyment
Hwang et al [24], 2024	To deliver biomechanical EMS for lower-limb virtual reality tasks	Quadriceps, tibialis anterior, hamstrings, gastrocnemius	9 (system validation), 12 (user evaluation)	Haptic force accuracy, sense of presence
Cheng et al [17], 2024	To enhance force feedback by EMS of antagonistic muscle pairs	Biceps, triceps	8 (formative study), 32 (user evaluation)	Preferences for realism, harmony, entertainment, comfort, overall

a N/A: not applicable.

### Balancing Performance Enhancement and Sense of Agency

Previous research suggests that enhancing performance while preserving the sense of agency is critical to effective EMS application. Performance enhancement is typically measured by preemptive gain over a user’s natural response [6]. The sense of agency refers to the neural mechanisms underlying an individual’s awareness of initiating, executing, and controlling voluntary actions [7,26,27]. One of the main challenges of EMS use is the potential disruption of agency [1,6], causing users to perceive their movements as externally controlled [5]. Kasahara et al [6] identified a stimulation “sweet spot” that enables users to feel the initiated movement voluntarily, achieving performance gains and moderate preservation of agency. These findings warrant further exploration in applied contexts.

Overstimulation can induce fatigue or impair voluntary motor control, whereas suboptimal stimulation may yield limited benefits [28]. Striking the right balance is critical, as neural adaptations influence how the brain and muscles respond to external stimuli [14]. When delivered at the sweet spot, EMS can promote voluntary contractions without overriding sensory-motor systems, supporting efficient neural adaptation over time [29].

This balance is essential in practical applications—such as entertainment, training, or rehabilitation—where significant preemptive gain must be achieved without cognitive dissonance. Attaining the sweet spot facilitates immediate performance gains and supports long-term improvements in strength, motor control, and functional capacity [14,30]. However, to our knowledge, no studies have applied this concept in realistic game-based scenarios.

### Rationale for Personalization Approaches

Research indicates that personalized EMS settings—adjusted for pulse width, stimulation timing, and electrode placement—produce more efficient and reliable outcomes [10,11,31]. Kono et al [9] emphasized the value of multichannel EMS systems for precise control of distinct muscle groups. Gerovasili et al [12] demonstrated that EMS efficacy depends on muscle mass and neuromuscular condition, whereas Kemmler et al [13] reported considerable variability in muscle adaptation and strength. These findings underscore the importance of personalization, given the substantial influence of individual physiological and neural differences. Variations in baseline motor responses, muscle composition, and nervous system plasticity render standardized EMS approaches inadequate.

Personalized EMS minimizes the risk of fatigue and overstimulation, enabling longer, more effective sessions while maintaining stimulation within the user’s optimal tolerance range [10]. This is essential for practical applications, where prolonged EMS use must remain effective without causing discomfort, injury, or reduced motivation. Tailored stimulation also supports long-term neural adaptation [5,14], as users are more likely to achieve sustained improvements in motor control when EMS is aligned with their individual characteristics.

## Methods

### Study 1: Determination of Embodied EMS

#### Overview

To understand the effect of embodied EMS, determining the EMS timing corresponding to the sweet spot and comparing the effect of embodied EMS across various conditions are necessary. Therefore, in Study 1, we determined the average of embodied EMS, and this average was used for Study 2.

#### Proposed Embodied EMS System Flowchart

**Figure 1.**
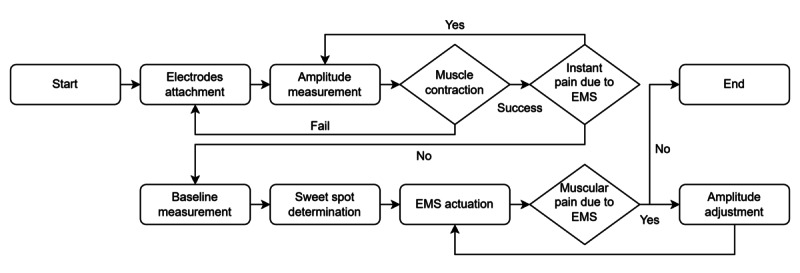
Flowchart of proposed embodied electrical muscle stimulation (EMS) system. The stages include calibration, sweet spot determination, EMS actuation, and user feedback.

The embodied EMS system comprises calibration, baseline measurement, sweet spot determination, and user feedback. Initially, electrodes are attached to the target position. Calibration involves setting the individualized EMS amplitude, a parameter crucial for inducing muscle contraction. This personalization is necessary because of interuser variability in appropriate stimulation levels [12,13,25]. If the user does not perceive stimulation, electrodes are repositioned. Conversely, if stimulation is perceived, amplitude is increased until muscle movement is induced without pain, at which point calibration concludes. The sweet spot determination stage identifies the optimal balance between preemptive gain and sense of agency. An elevated agency level may result in minimal preemptive gain, whereas excessively rapid preemptive gain can significantly diminish the sense of agency. Thus, the objective is to establish a moderate agency level. Following sweet spot determination, practice sessions commence with an actual task, enabling detailed adjustments based on user feedback. Participants complete a limited number of trials to confirm consistent muscle induction. Should muscle contraction be intermittent or pain reported, electrode placement is adjusted, and the process reverts to the calibration stage. Figure 1 shows the complete embodied EMS system flowchart.

#### Ethical Considerations

All experimental protocols were approved by the Hanyang University Institutional Review Board (case number: HYUIRB-202304‐022). All participants received a comprehensive overview of the procedure and provided written consent, and their data were deidentified. In appreciation of their involvement, all participants received compensation of $20 per hour. Individual users were not identified in any images of this study.

#### Participants

A total of 13 participants (5 women and 8 men) were recruited for this study. The participants had a mean age of 25.76 years (SD=3.65). All the participants were right-handed. Recruitment was conducted via bulletin boards on campus and the university website.

#### Hardware and Software

**Figure 2.**
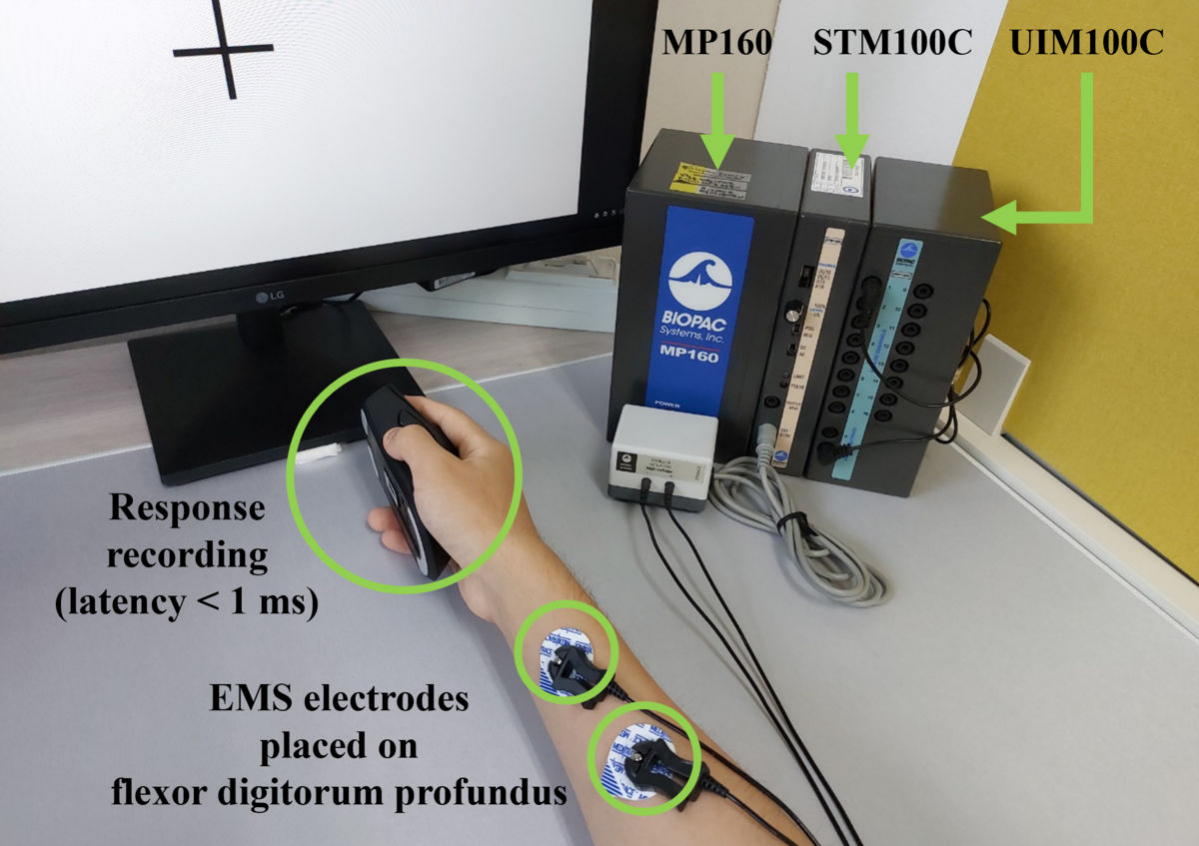
Experimental settings for Study 1. Electrical muscle stimulation (EMS) was controlled by a stimulation system (MP160, STM100C, and UIM100C). A pair of electrodes was placed on the participants’ forearms. Participants respond to the visual stimulus using a wireless gaming mouse.

Figure 2 shows the experimental setup. For EMS control, we utilized the MP160, STM100C, and UIM100C systems (Biopac Systems Inc., USA). AcqKnowledge (Biopac Inc., USA), software specifically designed for Biopac modules, facilitated EMS control and communication between the modules and the experimental program. A pair of electrodes was attached to the flexor digitorum profundus [6] to guide muscle contraction. The flexor digitorum profundus is a compartmental, multitendinous muscle [5,6,32] located in the forearm. It serves as the sole flexor of the distal interphalangeal joint, working in conjunction with other extrinsic (flexor digitorum superficialis and extensor digitorum) and intrinsic hand muscles to control various hand and finger movements, such as grasping and object manipulation. Despite being a single muscle, it comprises 4 compartments capable of relatively autonomous activation at low force levels to flex each fingertip [33].

The experimental program was developed using Unity 2022.3.5f1 (Unity Technologies, USA) and executed on a PC running Windows 10 Home edition (64-bit OS, Microsoft, USA). The system was powered by a Core i5-9600KF processor (3.70 GHz) (Intel Corporation, USA), 32 GB RAM (Samsung, South Korea), and a GeForce GTX 1660 Ti graphics card (NVIDIA, USA).

Participant responses (right-button clicks) to the cues were captured using a DeathAdder V2 Pro wireless gaming mouse (Razer Inc., USA) with a latency of 1 ms. Grip tape was applied to prevent hand slipping, and a supportive structure was used to maintain proper hand posture.

The response time of the system was from the moment of visual stimulus presentation command to the participant’s muscle response, and it approximately took 45 ms. It included 5 sequential steps. In step 1, the Unity activates a trigger for the AcqKnowledge software. In step 2, AcqKnowledge activates the EMS trigger. In step 3, the EMS is delivered to the participant’s forearm. In step 4, the EMS induces muscle contraction and leads to mouse clicks. In step 5, the Unity detects the mouse click and sends the termination command.

#### Calibration of EMS

We calibrated the EMS parameters [6,10,11], using a single, symmetric biphasic square wave with a 4-ms pulse width. This pulse width was determined through internal testing to be optimal for inducing precise finger movements. We opted against a fixed amplitude due to its high dependency on individual pain tolerance [6,10], instead of employing an individualized calibration approach. The amplitude, measured in volts, was iteratively calibrated for each participant through a 3-step process, with a 3-minute rest period between each step.

First, the adaptation phase prevented participants from startling. In total, 2 electrodes were attached to the participant’s forearm, and a single EMS pulse (4-ms pulse width) was applied. The amplitude gradually increased from 0, ensuring no stimulation caused pain or discomfort. The maximum amplitude at which participants reported no pain or discomfort after several repetitions was recorded as the critical value. The same process was then repeated with continuous EMS, incorporating a 3-ms interpulse interval and a 3-second stimulation duration, also starting from 0 amplitude.

Second, electrode positions were adjusted to ensure the EMS reliably actuated or produced a greater response in the middle finger than in any other part of the hand. A single EMS pulse at the critical amplitude, determined in the adaptation phase, was used. Participants were instructed to adjust their arm and palm angles to optimize middle finger activation.

Finally, participants held the mouse in the optimal position established in the previous step. A single EMS pulse at the critical amplitude was applied to ensure immediate, involuntary actuation of the middle finger, resulting in a right-button click. The amplitude was fine-tuned as needed based on participant response and the degree of middle finger activation.

#### Sweet Spot Determination: Visual Stimulus-Response Task

**Figure 3.**
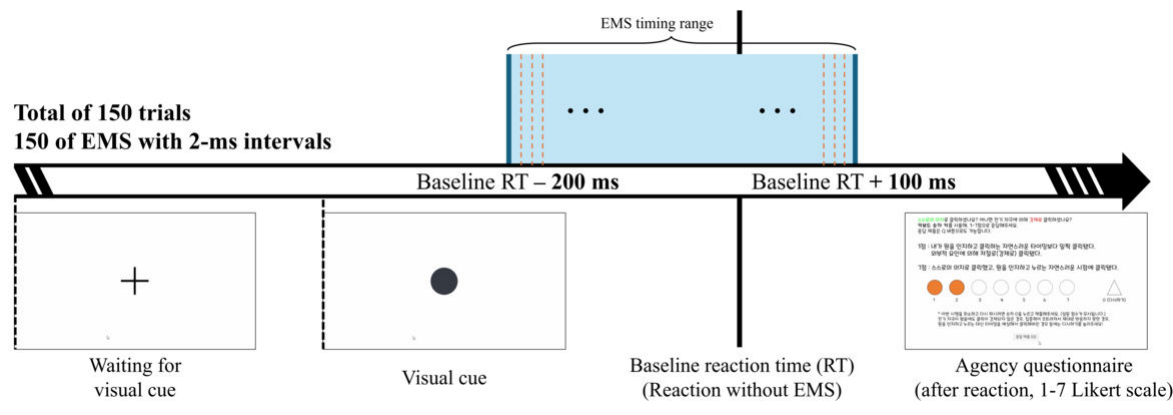
Visual stimulus-response task for sweet spot determination. A baseline of reaction time, which is response ability without electrical muscle stimulation (EMS), was measured first. The timing range of EMS was set by the individual baseline. Subsequently, the participants engaged in 150 trials of the task with the sense of agency questionnaire.

The task was a standard reaction-time measurement test (Figure 3). Participants were instructed to click the right button of the mouse upon recognizing the visual cue represented by a circle. Following each reaction, the participants were presented with a Likert scale questionnaire (ranging from 1 to 7) to assess their perceived sense of agency. The scale ranged from 1, meaning “I did not press the right button to click,” to 7, meaning “I pressed the right button to click.” Participants were asked to rely on their intuition when responding to the questionnaire.

The EMS actuation timing ranged from −200 to +100 ms based on the participant’s reaction time without the EMS (baseline). For example, if a participant had a baseline reaction time of 250 ms, the EMS timing window would range from 50 to 350 ms after the visual cue. We assigned 150 points with 2-ms intervals into 5 blocks, ensuring no redundancy across the blocks. To minimize the learning effect, the order of 30 points within each block was randomized.

#### Procedures

Before engaging in the main task, the participants completed a practice phase of up to 10 trials. Subsequently, the participants were asked to perform 30 trials without the EMS to establish their baseline reaction times. The participants completed the visual stimulus-response task. The task was to perform 150 right-button clicks on the target using an experimental mouse with timing ranging from −200 to +100 ms. The EMS was administered at a moment within the designated time window in every trial. Each trial was conducted using an agency questionnaire. The participants were debriefed after completing the trials.

#### Statistical Analysis

The relationship between preemptive gain and sense of agency (sweet spots of each participant) was calculated using logistic regression. Linear regression between EMS offset time and reaction time was conducted using the IBM SPSS 27.0 (SPSS Inc., USA) software package. Statistical significance was set at *P*<.05.

Before the analysis, we normalized the data by subtracting each participant’s baseline reaction time from each reaction time (with EMS actuation). This allows us the time gained by means of preemption (preemptive gain). We then normalized the agency axis from 0 to 1 and the horizontal axis to depict −400 to 400 ms. Logistic regression computes the relationship between the perceived agency and the preemptive gain per participant.

### Study 2: Evaluation of the Effect of Embodied EMS in a Practical Game for Physical Training

#### Overview

Based on the results of Study 1, we investigated the effects of EMS in a practical game scenario. We employed a pistol-shooting game that required fast reactions and cognitive judgment (ie, whether to act or not). Study 2 examined the effects of embodied EMS and additional effects of individual approaches. The task was conducted under 4 distinct conditions. The averagely embodied EMS condition used the average sweet spot from the results of Study 1, whereas the individually embodied EMS condition used the sweet spot from individual measurements using a method suggested in Study 1. In the first baseline condition (immediate EMS), the EMS was delivered immediately upon the appearance of the target. The second baseline condition (no EMS) enabled us to record participants’ natural reaction times. By comparing performance across these conditions, we assess how different EMS settings influence outcomes.

#### Ethical Considerations

We employed the same ethical considerations as Study 1.

#### Participants

A total of 10 participants (3 women and 7 men) who did not participate in Study 1 were included. The participants had a mean age of 24.44 years (SD=3.71), and all were right-handed.

#### Hardware and Software

We employed the same experimental setting as Study 1.

#### Sweet Spot Determination: Visual Stimulus-Response Task

Before beginning the pistol-shooting training, participants underwent a measurement described in Study 1 to identify individual sweet spots for the individually embodied EMS condition.

#### Evaluation Task: Pistol-Shooting Task

**Figure 4.**
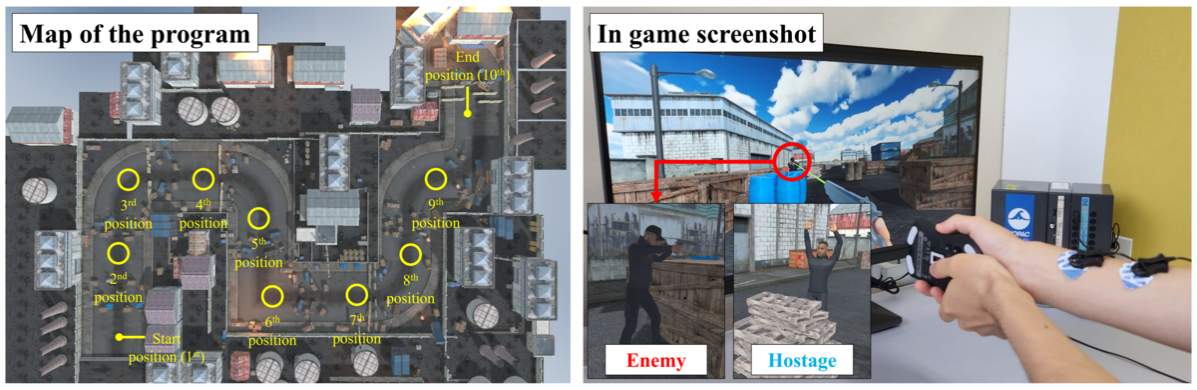
Pistol-shooting game with electrical muscle stimulation (EMS). Participants engaged in 4 conditions: averagely embodied EMS (EMS timing was fixed with an average sweet spot from Study 1), individually embodied EMS (EMS timing with an individualized sweet spot), immediate EMS (EMS timing presented when the enemy agent appears), and no EMS (participants respond without EMS).

We used a pistol-shooting program [34] similar to Virtua Cop 2 (SEGA Games, Japan) (Figure 4). In the game, participants were tasked with reaching a designated location, distinguishing enemy targets from hostages, and firing at the enemies. A target board displaying “start” appeared in the virtual space before the game began. The game began when the participant hit the target. Upon reaching the shooting position, 10 agents—comprising 4-6 hostages and 6-4 enemies—appeared (correspondingly) with random jittering time (from 500 to 1500 ms). Participants had to eliminate all enemies at the given location to proceed to the next position. The targets at each position were arranged in a 10×1 (horizontal×vertical) spherical coordinate system, with a horizontal range of −55° to +55°, and each target was spaced at a 10° radial distance. In total, 10 shooting positions were considered; 50 enemies and 50 hostages were presented, accompanied by corresponding sound effects.

#### Procedures

After participants were briefed, personalized values for the individually embodied EMS conditions were measured as in Study 1. In the second visit, participants engaged in a practice session of the pistol-shooting program. The pistol-shooting task was performed at least 3 days after the first visit to minimize the learning effect. Participants did this task under 4 conditions: averagely embodied EMS, individually embodied EMS, immediate EMS, and no EMS, presented in counterbalanced order. During the task, participants completed a sense of agency questionnaire. After the experiment, participants were debriefed.

#### Statistical Analysis

The relationship between preemptive gain and sense of agency (individual “sweet spots”) was calculated using logistic regression, as in Study 1. Owing to variability in subjective feelings and physical conditions, participants were categorized into 2 subgroups for further analysis based on their measured sweet spots: those with sweet spots faster than average (FTA group) and those slower than average (STA group). We then investigated whether the effects of the 4 EMS conditions differed between these 2 groups.

All statistical analyses were performed using SPSS 27.0 (SPSS Inc., USA). Normality, skewness, kurtosis, and Shapiro-Wilk tests were conducted. Sphericity was assessed using the Mauchly test; if violated, the Greenhouse-Geisser correction was applied. A mixed ANOVA investigated the differences between the FTA and STA groups across the 4 EMS conditions: averagely embodied EMS, individually embodied EMS, immediate EMS, and no EMS. Statistical significance was set at *P*<.05. Post hoc analyses for group differences and comparisons across the 4 EMS conditions both utilized paired t tests. Data from 9 participants were used in the analysis, as 1 female participant’s data were excluded because of a calibration failure.

## Results

### Results of Study 1

In total, 1950 trials were collected from all participants, with 2 data points (reaction time and sense of agency) recorded per trial. The average baseline was 238.3 ms (SD=29.2 ms), which aligns with findings in psychophysics research that report an average reaction time of 250 ms in response to visual stimuli [6,35]. The baseline reaction time for the fastest and slowest participants was 212.8 and 304.8 ms, respectively. Consequently, the EMS offset timing ranged from 12.8 to 402.8 ms after the onset of the visual stimulus. Table 2 presents the measurement results for each participant.

**Table 2.** Measurement results for Study 1^a^.

Participant	Sex	Stimulus timing (ms)	Baseline (ms)	Sweet spot (ms)
1	Male	31.0‐329.0	231.0	68.2
2	Male	13.6‐311.6	213.6	20.1
3	Male	12.8‐310.8	212.8	17.4
4	Male	27.4‐325.4	227.4	38.8
5	Male	85.9‐383.9	285.9	28.1
6	Female	13.1‐311.1	213.1	−4.0
7	Female	19.1‐317.1	219.1	121.7
8	Male	58.8‐356.8	258.8	28.1
9	Male	41.1‐339.1	241.1	57.5
10	Female	16.1‐314.1	216.1	28.1
11	Male	27.4‐325.4	227.4	84.3
12	Female	104.8‐402.8	304.8	20.1
13	Female	46.4‐344.4	246.4	44.1

a All participants first measured baseline response time to visual stimuli in the absence of EMS. Based on the individually measured baseline, stimulus timings ranging from −200 to +100 ms EMS were determined. Afterward, the sweet spot was calculated.

**Figure 5.**
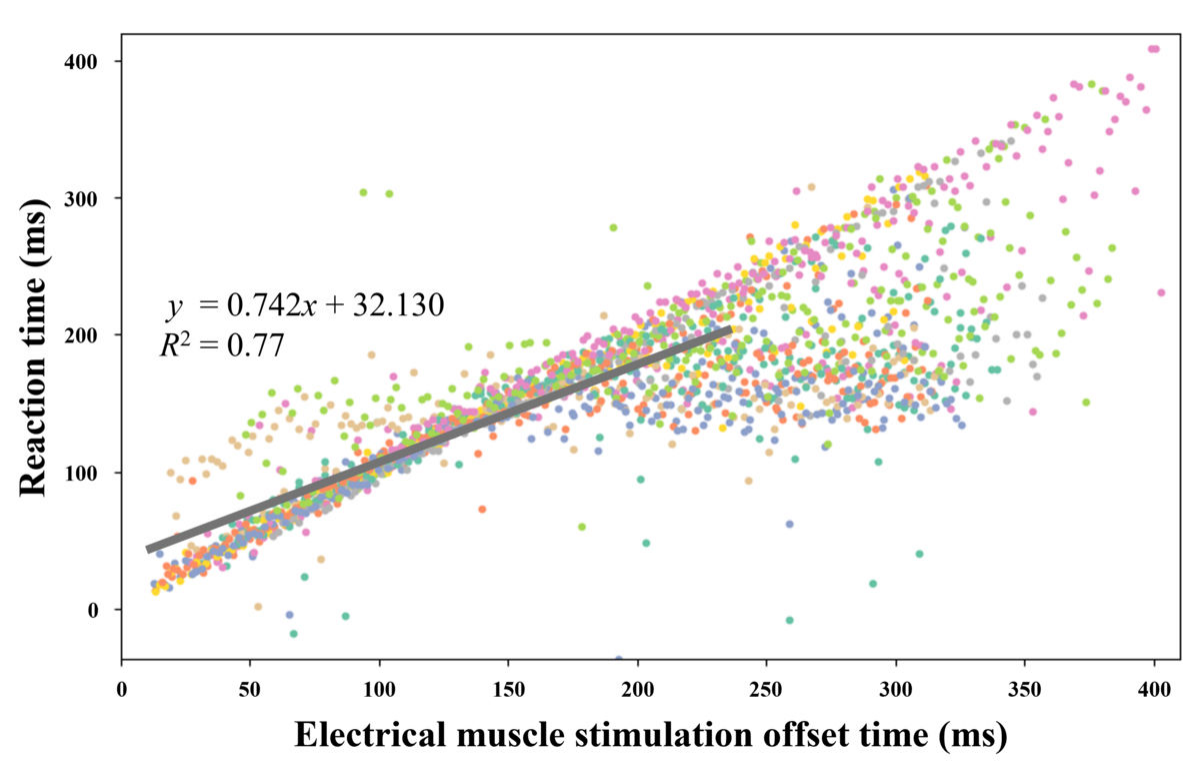
Relationship between electrical muscle stimulation (EMS) offset time and reaction time. The linear relationship between EMS timing and reaction time in the area within the gray line suggests that the EMS directly induced the reaction. The values in the area beyond the gray line are dispersed because the EMS was applied after the natural reaction speed.

EMS actuation reduced reaction time. Figure 5 shows the relationship between the EMS offset and total reaction time. This relationship was linear between 12.8 ms (the earliest reaction time influenced by EMS) and 238.3 ms (the average reaction time), indicating that EMS increased the reaction times.

**Figure 6.**
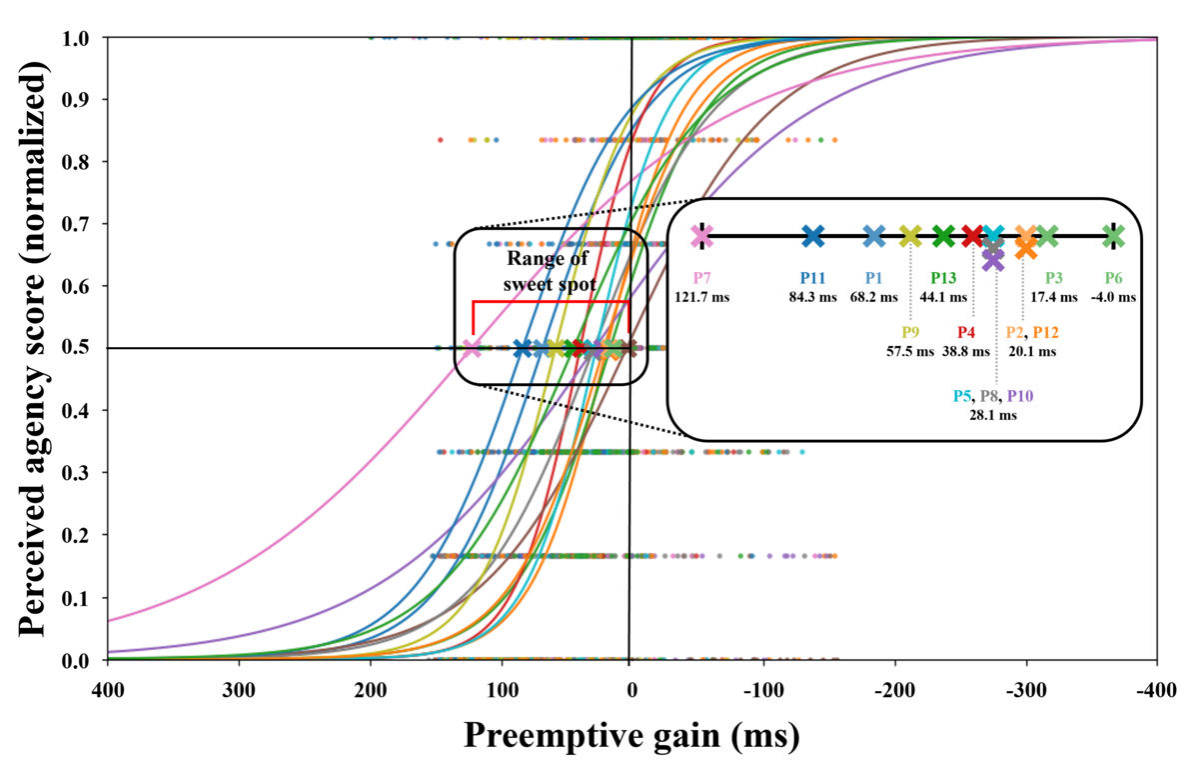
Result of logistic regression analysis between preemptive gain and sense of agency in Study 1. The average sweet spot for the 13 participants was 46.8 ms.

Through logistic analysis, the average sweet spot for all participants was 46.8 ms. This general sweet spot was used as the averagely embodied EMS condition in Study 2. We also found the individual differences of those sweet spots. The highest level of preemptive gain among participants was 121.7 ms, whereas the lowest preemptive gain was −4.0 ms. Figure 6 shows the logistic regression analysis for each participant, indicating the relationship between agency and preemptive gain.

### Results of Study 2

#### Results of Individual Sweet Spot Measurement

We collected 1350 trials from participants, with 2 data points (reaction time and sense of agency response) recorded per trial. The average baseline of Study 2 was 236.8 ms (SD=21.0 ms), which is consistent with the findings from Study 1 (average=238.2 ms) and previous research [6,35]. The baseline reaction time for the fastest and slowest participants was 206.9 and 262.7 ms, respectively. Table 3 presents the measurement results for each participant.

**Figure 7.**
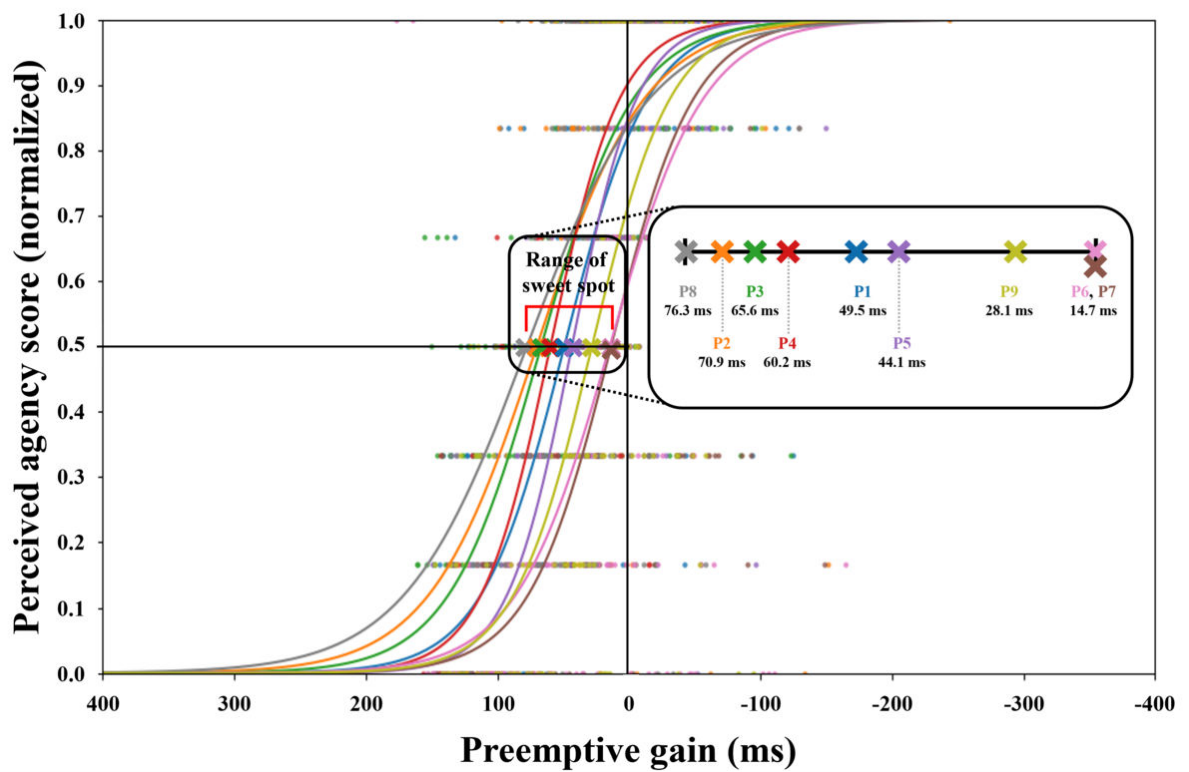
Result of logistic regression analysis between preemptive gain and sense of agency in Study 2.

Using logistic analysis, we calculated the sweet spots for each participant in Study 2. The average sweet spot for all participants was 54.8 ms. The levels of preemptive gain for the fastest and slowest participants were 76.3 and 14.7 ms, respectively. Figure 7 shows the logistic regression analysis for each participant, indicating the relationship between agency and preemptive gain.

**Table 3.** Measurement results of Study 2^a^.

Participant	Sex	Baseline (ms)	Sweet spot (ms)	Group
1	Female	262.7	49.5	FTA^b^
2	Male	208.0	70.9	FTA
3	Male	225.6	65.6	FTA
4	Male	252.3	60.2	FTA
5	Male	259.5	44.1	STA^c^
6	Male	243.6	14.7	STA
7	Male	206.9	14.7	STA
8	Female	246.8	76.3	FTA
9	Male	226.1	28.1	STA

a All participants first measured baseline response time to visual stimuli in the absence of electrical muscle stimulation (EMS). Based on the individually measured baseline, stimulus timings ranging from −200 to +100 ms EMS were determined. Subsequently, the sweet spot was measured. If the sweet spot was faster than the average value of Study 1, the participants were assigned to the FTA group, and if it was slower, the participants were assigned to the STA group.

b FTA: faster than average.

c STA: slower than average.

#### Response Time

A significant main effect of the EMS conditions (*F* (1.805, 14.437)=27.528, *P*<.001, *η*^2^=0.79) was observed. However, no significant main effect of the group and interaction effect (*F*_1,7_=0.175, *P*=.69, *η*^2^=0.024; *F*_1.856, 12.989_=0.554, *P*=.58, *η*^2^=0.073, respectively) was observed. Table 4 shows response time analysis results in each EMS condition and group.

The averagely embodied EMS condition was slower than the immediate-EMS condition (*t*_8_=3.881, *P*=.03) and faster than the no-EMS condition (*t*_8_=3.776, *P*=.04). No significant difference was observed between the individually embodied and averagely embodied EMS conditions (*t*_8_=–0.986, *P*>.99). The response time in the individually embodied EMS condition was also slower than that in the immediate-EMS condition (*t*_8_=5.792, *P*=.002) and faster than that in the no-EMS condition (*t*_8_=6.753, *P*=.001). The response time in the immediate-EMS condition was significantly shorter than that in the no-EMS condition (*t*_8_=–7.970, *P*=.001).

**Table 4.** Results of the response time^a^.

Response time, mean (SD)	Averagely embodied EMS	Individually embodied EMS	Immediate EMS (baseline 1)	No EMS (baseline 2)
Faster than average (FTA) group	202.1 (38.7)	218.2 (54.4)	131.5 (79.0)	273.5 (63.8)
Slower than average (STA) group	209.6 (22.3)	214.0 (29.4)	96.7 (44.8)	259.0 (41.5)

a A total of 4 electrical muscle stimulation (EMS) conditions were included: averagely embodied EMS, individually embodied EMS, immediate EMS, and no EMS. Participants were grouped into 2 groups: the faster than average (FTA) group and slower than average (STA) group.

#### Sense of Agency

Significant main effects of the EMS condition and interaction effects between the EMS condition and groups (*F*_3, 24_=63.350, *P*<.001, *η*^2^=0.900; *F*_3, 21_=3.786, *P=*.03, *η*^2^=0.351, respectively) were observed. However, no significant difference was observed depending on the group (*F*_1, 7_=1.753, *P*=.23, *η*^2^=0.200). Table 5 shows the sense of agency analysis results in each EMS condition and group.

**Table 5.** Results of the sense of agency^a^.

Response time, mean (SD)	Averagely embodied EMS	Individually embodied EMS	Immediate EMS (baseline 1)	No EMS (baseline 2)
Faster than average (FTA) group	4.7 (0.7)	4.1 (0.9)	3.0 (1.0)	6.7 (0.7)
Slower than average (STA) group	3.3 (1.6)	4.3 (0.9)	1.8 (0.4)	7.0 (0.0)

a A total of 4 electrical muscle stimulation (EMS) conditions were included: averagely embodied EMS, individually embodied EMS, immediate EMS, and no EMS. Participants were grouped into 2 groups: the faster than average (FTA) group and slower than average (STA) group.

Sense of agency in the averagely embodied EMS condition was higher than in the immediate-EMS condition (*t*_8_=5.807, *P*=.002) and lower than in the no-EMS condition (*t*_8_=5.736, *P*=.003). No significant difference was observed between the individually embodied and averagely embodied EMS conditions (*t*_8_=–0.315, *P*>.99). Sense of agency in the individually embodied EMS condition was also higher than in the immediate-EMS condition (*t*_8_=4.495, *P*=.01) and lower than in the no-EMS condition (*t*_8_=–7.419, *P*<.001). Sense of agency in the no-EMS condition was significantly higher than in the immediate-EMS condition (*t*_8_=–12.452, *P*<.001).

The sense of agency across the 4 conditions manifested differently in the 2 groups. In the FTA group, the averagely embodied EMS condition was higher than the immediate-EMS condition (*t*_4_=8.064, *P*=.001) and lower than the no-EMS condition (*t*_4_=–5.706, *P*=.005). Agency in the individually embodied EMS condition was significantly lower than in the no-EMS condition (*t*_4_=–4.466, *P*=.01). However, no significant difference was observed with immediate EMS (*t*_4_=2.125, *P*=.10). Agency in the immediate- and no-EMS conditions was significantly different (*t*_4_=–9.105, *P*=.001).

Unlike the FTA group, in the STA group, the sense of agency in the individually embodied EMS condition was significantly higher than in the immediate-EMS condition (*t*_3_=–8.996, *P*=.003) and significantly lower than in the no-EMS condition (*t*_3_=–6.063, *P*=.009). No significant difference was observed with the averagely embodied EMS condition (*t*_3_=2.284, *P*=.12). The averagely embodied EMS condition was not significantly different from the immediate-EMS condition (*t*_3_=2.414, *P*=.095). However, the agency was lower than the no-EMS condition (*t*_3_=–4.680, *P*=.02). Agency in the immediate-EMS was significantly lower than in the no-EMS condition (*t*_3_=–24.981, *P*<.001).

## Discussion

### Principal Findings

The findings offer valuable insights into the use of embodied EMS for enhancing rapid reaction performance in serious games and related contexts. Averagely and individually embodied EMS significantly improved response times over baseline conditions while preserving the sense of agency. Individually embodied EMS provided further benefits, highlighting the value of personalization in EMS applications.

Study 1 identified a specific EMS timing sweet spot that significantly improved reaction time while preserving the sense of agency. EMS applied within this window (average preemptive gain: 46.8 ms) enhanced responses without diminishing perceived control. Notable interindividual variability highlighted the need for personalized EMS settings, as uniform configurations may yield suboptimal results owing to physiological differences.

Study 2 applied these findings to a practical pistol-shooting game requiring rapid and accurate reactions. Averaged and individually calibrated EMS significantly reduced reaction times, confirming that the identified sweet spot improves physical response in applied contexts. Individually embodied EMS provided additional benefits, reinforcing the value of personalization. This aligns with prior research [5,6] showing that customized EMS enhances performance while minimizing fatigue.

The comparative analysis between the FTA and STA groups revealed important individual differences. Both groups reported an intermediate sense of agency under the individually embodied EMS condition, indicating that the sweet spots were effectively personalized. However, differences in agency levels between the groups suggest variation in how participants perceived control over their actions. Although sweet spot calibration was conducted using a nonjudgmental task, it was subsequently applied to a pistol-shooting game involving decision-making (ie, whether to shoot or not), introducing additional cognitive load. This may explain why participants in the FTA group reported a stronger sense of agency in the averagely embodied EMS condition. In contrast, the STA group appeared particularly sensitive to the sense of agency under individually embodied EMS and exhibited markedly low agency scores in the immediate-EMS condition. This suggests heightened sensitivity to externally induced control and a stronger aversive reaction to forced interventions.

Additionally, the STA group may require more time to form a sense of agency. Their average sweet spot was 25.4 ms, compared to 64.5 ms in the FTA group, indicating that their reaction speed improved only by 39.4% relative to the FTA group. Despite this discrepancy, both groups reported a similar sense of agency under the individually embodied EMS condition, suggesting that the STA group, while showing smaller performance gains, achieved comparable subjective control—likely through more gradual interventions.

The findings from Study 2 underscore the importance of personalized EMS settings in practical applications. Participants exhibited considerable variability in optimal stimulation timing, and the impact of individually embodied EMS differed across groups. Thus, applying a uniform average sweet spot is insufficient. Instead, tailoring EMS to individual profiles is essential to maximize performance benefits and maintain user agency.

### Comparison to Prior Works

This study extends the work of Kasahara et al [5,6] by demonstrating how agency-preserving EMS can enhance motor performance in more ecologically valid scenarios. Although prior studies employed simplified reaction-time tasks (eg, tapping in response to a visual cue), we applied EMS in a dynamic pistol-shooting game requiring visual discrimination and decision-making. This context more closely reflects real-world applications and cognitive demands. Furthermore, we refined the concept of the “sweet spot” by introducing individualized EMS timing rather than relying on fixed or averaged values. This personalization accounts for interindividual variability in agency perception and motor responsiveness, and our findings suggest that tailored EMS delivery offers additional performance and agency-related benefits. Finally, we present a practical system pipeline—from EMS calibration to real-time actuation and feedback—providing a replicable framework for deploying EMS in interactive applications.

Beyond reaction enhancement, prior research has shown that EMS can improve muscle strength and posture, illustrating its broader applicability [24,25]. Strojnik et al [18] and Takahashi et al [3] demonstrated that EMS can effectively target specific muscle groups to support motor function improvements. These findings indicate that EMS-enhanced training can be extended to muscle conditioning and injury prevention, in addition to reflex-based tasks. Integrating EMS into structured strength and rehabilitation programs may provide neuromuscular reinforcement, enhance postural control, and improve performance during high-intensity movements. This also aligns with rehabilitation protocols where EMS has been used successfully to restore muscle function in patients recovering from injury [11,12]. By incorporating EMS for performance enhancement and injury prevention, athletic training programs and professional organizations can leverage its potential to support short-term gains and long-term physical resilience.

The individually embodied EMS system demonstrated its capacity to enhance motor performance within a game-based context, and this capability can be extended to a range of real-world applications. Athletes in high-speed disciplines—such as fencing or shooting—require rapid reflexes and high levels of precision. The individually embodied EMS can be calibrated to improve reaction times during training, thereby enhancing competitive performance without compromising the athlete’s sense of control. This aligns with findings in strength training research, where EMS has been shown to increase maximal voluntary contraction through neural adaptations rather than muscle hypertrophy [14,28-30]. In high-stakes environments such as battlefield simulations or tactical decision-making tasks, the need for rapid responses and precise actions is equally critical. Our results suggest that individually embodied EMS can optimize reaction time in such contexts, potentially reducing errors in high-pressure scenarios. Notably, the ability to preserve a user’s sense of agency while enhancing speed is essential for maintaining situational awareness and cognitive control required in these fields.

Integrating findings from the current and prior research, we propose a 3-phase implementation roadmap for deploying EMS enhancements in real-world scenarios. First, a calibration phase should be conducted to identify each user’s individual sweet spot using nonjudgmental tasks, ensuring that stimulation improves response speed without disrupting the sense of agency. Second, EMS should be incorporated into structured training programs—such as athletic drills or immersive game-based environments—to develop reaction speed and motor precision. Third, a real-time feedback and adaptation phase should adjust EMS parameters dynamically based on the user’s performance and physiological state (eg, fatigue), thereby sustaining training effectiveness and reducing the risk of overstimulation. This roadmap offers a feasible pathway for implementing embodied EMS in commercial and professional settings, emphasizing personalization, adaptability, and user-centered design.

### Limitations and Future Works

Although this study offers valuable insights into the use of embodied EMS in practical scenarios, several limitations should be acknowledged. First, although our sample size was comparable to previous studies [5,6], it remained relatively small owing to the inherent challenges associated with EMS research. Future studies should replicate these findings with larger and more diverse participant populations to enhance generalizability. Second, although the pistol-shooting game was selected for its high demands on speed and precision, future research should investigate EMS applications in other complex, real-world tasks that require a balance between rapid responses and decision-making—such as surgical procedures or athletic competitions. Third, EMS in this study was applied to the flexor digitorum profundus, a commonly targeted muscle in EMS research [5,6,21,22]. However, more recent work has begun to explore EMS applications on other muscle groups [24]. Therefore, the effectiveness of embodied EMS across a wider range of muscles should be examined to determine its broader applicability. Finally, although this study highlighted the effectiveness of personalized EMS settings, it also underscored the need for more advanced EMS systems that can dynamically adapt to individual user conditions, such as fatigue or stress. Future research should optimize EMS systems to accommodate these fluctuations, ensuring sustained performance improvements across varying contexts.

Beyond immediate performance gains, an important question remains: Does EMS-based reaction training produce lasting improvements? Previous research offers some promising insights. Kasahara et al [5] reported that EMS training can yield long-term improvements in reaction time, even after the EMS is removed—likely due to neuromuscular plasticity and enhanced motor learning. These results are consistent with other studies involving repeated electrical stimulation, which show strengthened sensorimotor integration and improved voluntary control over time [2,14]. These findings suggest that EMS training may offer enduring benefits beyond its immediate effects. To fully explore this potential, future studies should conduct longitudinal assessments of participants’ reaction performance over extended periods.

Although EMS offers significant advantages in reaction training and human augmentation, its ethical implications warrant careful consideration in future research. The induction of involuntary muscle movement raises concerns about user autonomy, informed consent, and potential misuse, particularly in competitive contexts [1,7,8]. Future studies should prioritize systems that allow users to maintain full control over EMS intensity and activation parameters, ensuring that all movements remain voluntary. The application of EMS in competitive gaming or professional sports introduces ethical and regulatory concerns. Organizations should establish clear guidelines, distinguishing between training enhancement and real-time performance augmentation. Previous research by Faltaous et al [1] indicated that user acceptance of EMS depends largely on the degree of control, highlighting the need for ethical design considerations. Overuse of EMS could lead to muscle fatigue, adaptation limitations, or cognitive overload. Future research should focus on safety thresholds. Developing ethical EMS guidelines and regulatory policies will ensure responsible adoption in athletics, gaming, and human augmentation fields.

### Conclusion

The results of this study demonstrate the potential of embodied EMS in practical game scenarios that require rapid reactions and precise decision-making. By identifying and applying individualized sweet spots, we enhanced participants’ performance without compromising their sense of agency. Moreover, the individually embodied EMS configuration provided an additional benefit. This study contributes to the broader field of EMS research by showing how embodiment-preserving stimulation can effectively augment human capabilities in realistic, performance-driven contexts.

## Supplementary material

10.2196/69330 Multimedia Appendix 1 Measurement data.

## Abbreviations

EMS: electrical muscle stimulation
FTA: faster than average
STA: slower than average
